# Advent of CRISPR Based Immunotherapy in Hematologic Malignancies

**Published:** 2018-03-30

**Authors:** RK Perez, R Chen, R Kang, AR Perez

**Affiliations:** 1 University of California, 505 Parnassus Ave, San Francisco, CA 94143, USA; 2 Weill Cornell Medicine, 1300 York Ave, New York City, NY 10021, USA

## Commentary

Hematologic malignancies encompass a wide array of disease including leukemia, lymphomas, myelodysplastic syndrome, and multiple myeloma. These malignancies result from bone marrow dysfunction that yields clinical entities ranging from smoldering pre-leukemia states to outright acute leukemia. Aside from representing a diverse set of hematologic disease, these malignancies are also prevalent and projected to account for eight to nine percent of all newly diagnosed cancers in 2018 [1]. When Thomas Hodgkin first described his namesake lymphoma in seven patients from Guy’s Hospital nearly 200 years ago, virtually all hematologic cancers were fatal. The nineteenth century English physician Thomas Fowler, who utilized arsenic in the treatment of leukemia, proposed one of the first uses of cytotoxic chemotherapy [2,3]. Similarly, Sidney Farber’s studies of aminopterin in children with acute lymphoblastic leukemia demonstrated the ability of chemotherapy to produce disease remission [4,5]. As oncology’s understanding of hematologic cancers progressed, so did the character of the field’s chemotherapeutics. When the Bcr-Abl chromosomal rearrangement was linked to the pathogenesis of chronic myelogenous leukemia, a new class of chemotherapy emerged: small molecule inhibitors [6,7]. The archetype of this class of chemotherapy is imatinib, a small molecule developed to specifically inhibit the action of the Bcr-Abl fusion kinase protein. The action of this small molecule effectively induces clinical, and potentially molecular, remission in patients with the fusion onco-protein [8,9]. With the success of imatinib, small molecule inhibitors garnered much excitement, which furthered research on their therapeutic potential. However, while the development of chemotherapeutics for hematologic cancers has advanced tremendously, much progress remains. One area in which the frontier of cancer treatment is being pushed is in multiple myeloma.

Multiple myeloma is the neoplastic proliferation of plasma cells in the bone marrow leading to extensive osteolytic lesions, osteopenia and anemia. Multiple myeloma accounts for one to two percent of all cancers and around seventeen percent of hematologic malignancies in the United States annually [1]. Multiple myeloma predominantly occurs in older adults with a median age at diagnosis of sixty-six [10]. The disease occurs slightly more frequently in men and evidence suggests it has a higher incidence in people of African American background compared to Caucasians and Asians [11,12]. Response to therapy is heterogeneous with some patients demonstrating treatment refractory disease while others experience lasting remission. The median overall survival rate for patients with multiple myeloma is 5.2 years [13]. All symptomatic patients undergo induction therapy unless toxicities are evident. Patients who undergo hematopoietic stem cell transplantation have been shown to have better survival outcomes than those who receive only chemotherapy [14,15]. To date, effective therapy for multiple myeloma is autologous stem cell transplantation with chemotherapy [16]. The therapy is not curative and it carries notable treatment associated mortality [16]. However, with the advent of CRISPR and its utilization in cancer immunotherapy, new therapies for multiple myeloma are actively being developed.

CRISPR technology has revolutionized the biomedical sciences [17,18]. The simplicity of type II CRISPR systems allows an investigator to edit virtually any element of a genome using a two-component system consisting of a CRISPR endonuclease and a guide RNA [19,20]. The regions in the genome that are amenable to editing by CRISPR are defined by an approximately three-base sequence—the protospacer adjacent motif (PAM), which is directly recognized by the CRISPR endonuclease [21]. To modify a specific segment of a genome an investigator needs to ensure that a PAM exists in the target region and then modify an approximately twenty-base region of a guide RNA such that it is complementary to the DNA adjacent to the PAM. Ensuring the precision of guide RNAs to a target site can be established using currently available bioinformatics software [22,23]. Together the CRISPR endonuclease and guide RNA target a specific genomic location and induce a double strand break. The cell typically repairs the double strand break through either non-homologous end joining (NHEJ) or homology-directed repair (HDR) [24]. NHEJ leads to variable length deletions or insertions and can lead to loss of function if used against gene exons [25]. HDR allows for the precise modification of a segment of DNA using exogenous donor DNA templates [25]. Combined, NHEJ and HDR repair mechanisms allow for an array of modifications to a genomic target region. The promise CRISPR editing technology holds for cancer research and treatment is immense. Perhaps nowhere is this promise more exciting than in CRISPR mediated immunotherapy for multiple myeloma.

Currently, a phase one clinical trial underway at the University of Pennsylvania utilizes CRISPR technology to edit patient T-cells in order to re-sensitize them towards multiple myeloma cells (NCT03399448). These T-cells undergo several CRISPR induced modifications to augment their efficacy as a therapeutic. The experimental therapy transduces autologous T-cells with a lentiviral vector expressing NY-ESO-1 T-cell receptor (TCR). NY-ESO-1 is a gene that has restricted expression in wild-type tissue but is frequently expressed in neoplastic tissue and found to be expressed in 37% of multiple myelomas [26,27]. Transducing T-cells with NY-ESO-1 TCR should sensitize them towards cancer cells expressing NY-ESO- 1. The T-cells then receive CRISPR guide RNAs to disrupt the expression of three endogenous genes: PD-1, TCRα and TCRβ. PD-1 is a transmembrane protein expressed on T-cells and B cells that binds to the PD-L1/2 ligand found on many tumor cells that directly inhibits apoptosis, promotes T effector cell exhaustion, and enhances conversion of T-effector cells into Treg cells [28,29]. By disabling the PD-1 gene, the investigators seek to prevent T-cell inactivation by cancer cells. Disrupting endogenous T-cell receptor (TCR α/β) expression is important to prevent off-target activity of the modified T-cells [30]. These CRISPR modified T-cells are designed specifically to target NY-ESO-1 cancer cells and be immune to inactivation by the cancer cell. Although adverse side effects for this therapy are unknown due its nascent stage, other similar therapies have observed cytokine release syndrome (CRS) in some patients [30]. CRS is a constellation of inflammatory symptoms resulting from elevated cytokine levels linked to T-cell activity and proliferation [31]. In most patients, the symptoms are flulike however severe and acute onset of CRS can be fatal. Some argue that CRS is likely a necessary consequence of T-cell activation and the use of corticosteroids to mitigate CRS reduces the efficacy of the therapy. Some suggests an IL-6 blockade by tocilizumab is effective at reversing CRS while having limited effect on T-cell efficacy [31].

Throughout the history of oncology, the treatment of hematologic malignancies foreshadowed the trajectory of cancer treatment as a field. CRISPR based immunotherapy has the potential to revolutionize how we treat and understand not only hematologic cancers, but all cancers. It holds the promise of being the new frontline of chemotherapy and allow for the personalization of cancer treatment.
